# Bilateral anterior ischemic optic neuropathy and choroidal ischemia in a patient with COVID‐19 infection

**DOI:** 10.1002/ccr3.6834

**Published:** 2023-01-23

**Authors:** Seyed Hossein Ghavami Shahri, Mojtaba Abrishami, Helia Shayanfar, Sahel Khazaei

**Affiliations:** ^1^ Eye Research Center Mashhad University of Medical Sciences Mashhad Iran

**Keywords:** angle‐closure glaucoma, anterior ischemic optic neuropathy, choroidal ischemia, SARS‐CoV‐2

## Abstract

A 57‐year‐old male known case of diabetes mellitus presented with gradually bilateral decreased vision accompanied by ocular pain two weeks after diagnosis of SARS‐CoV‐2 infection. The results of examination and imaging were indicative of bilateral anterior ischemic optic neuropathy (AION) and massive choroidal ischemia, which may be associated with SARS‐CoV‐2‐induced damages, including endothelial damage, hypercoagulable state, and hypoxia.

## Graphical abstract

The purpose of this report was to illustrate the occurrence of bilateral ischemic optic neuropathy and choroidal ischemia following COVID‐19. We aimed to shed light on the potential and multiplicity of ophthalmic presentation in a patient with COVID‐19 infection.

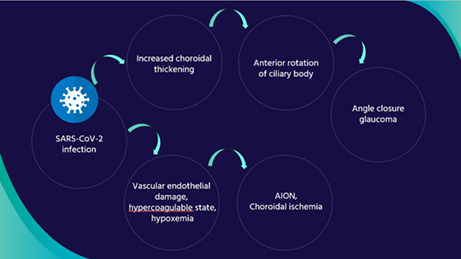

## 1 INTRODUCTION

Severe acute respiratory syndrome coronavirus 2 (SARS‐CoV‐2) is a highly contagious enveloped RNA virus that caused a global pandemic in December 2019. Although Coronavirus Disease 2019 (COVID‐19) was primarily found to affect the respiratory and gastrointestinal system, further studies revealed the multiorgan invasion of the virus.^1^ Although the spectrum of ocular manifestations is not completely defined, several reports from anterior segment involvement such as conjunctival hyperemia, chemosis, increased secretions, anterior uveitis to retinitis, choroiditis, and optic neuropathy are available.^2^, ^3^ The SARS‐CoV‐2 infection may be asymptomatic or paucisymptomatic with mild‐to‐moderate presentation of disease, and some individuals may present severe to critical disease. Therefore, ophthalmologists may encounter asymptomatic or paucisymptomatic individuals who presented ophthalmic manifestation of COVID‐19 infection.

Herein, we present bilateral nonarteritic anterior ischemic optic neuropathy (NAION) and choroidal ischemia in a patient with confirmed SARS‐Cov‐2 infection.

## 2 CASE DESCRIPTION

A 57‐year‐old man with a past medical history of controlled diabetes mellitus (DM), and no prior ophthalmic examination was referred due to gradually bilateral vision loss six days before the presentation which was accompanied by ocular pain in recent days. Eighteen days before the onset of visual symptoms, the patient was diagnosed with SARS‐CoV‐2 by a positive real‐time reverse transcription polymerase chain reaction (RT‐PCR) of a nasopharyngeal swab specimen and symptoms of high fever, persistent cough, and shortness of breath. The patient was admitted for two weeks and received systemic Dexamethasone and Remdesivir. At presentation, his viral and systemic symptoms were resolved. There was no history of headache, scalp tenderness, fever, weight loss, and muscle weakness.

**FIGURE 1:**
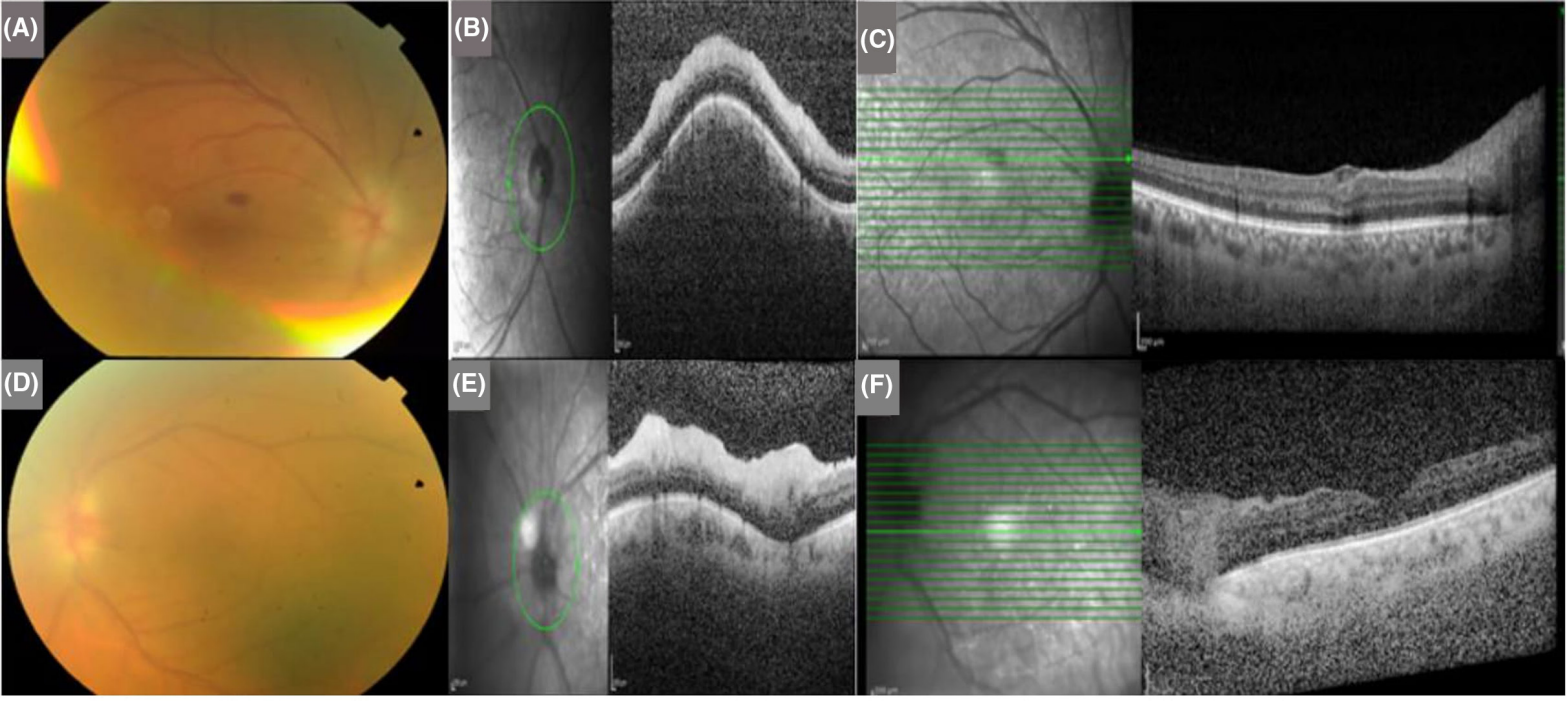
(A, D) Color fundus photography: Bilateral peripapillary retinal swelling and macular hemorrhage in the RE. (B, E) Retinal nerve fiber layer OCT: Bilateral peripapillary retinal swelling. (C, F) Enhanced depth imaging OCT: increased choroidal thickness in both eyes.

**FIGURE 2:**
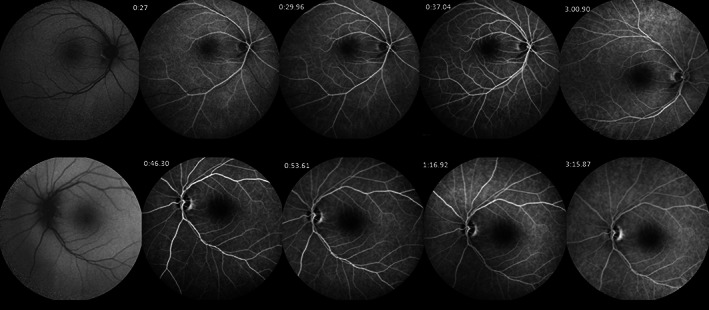
Fluorescein angiography: No evidence of areas of ischemia or peripheral vasculitis.

**FIGURE 3:**
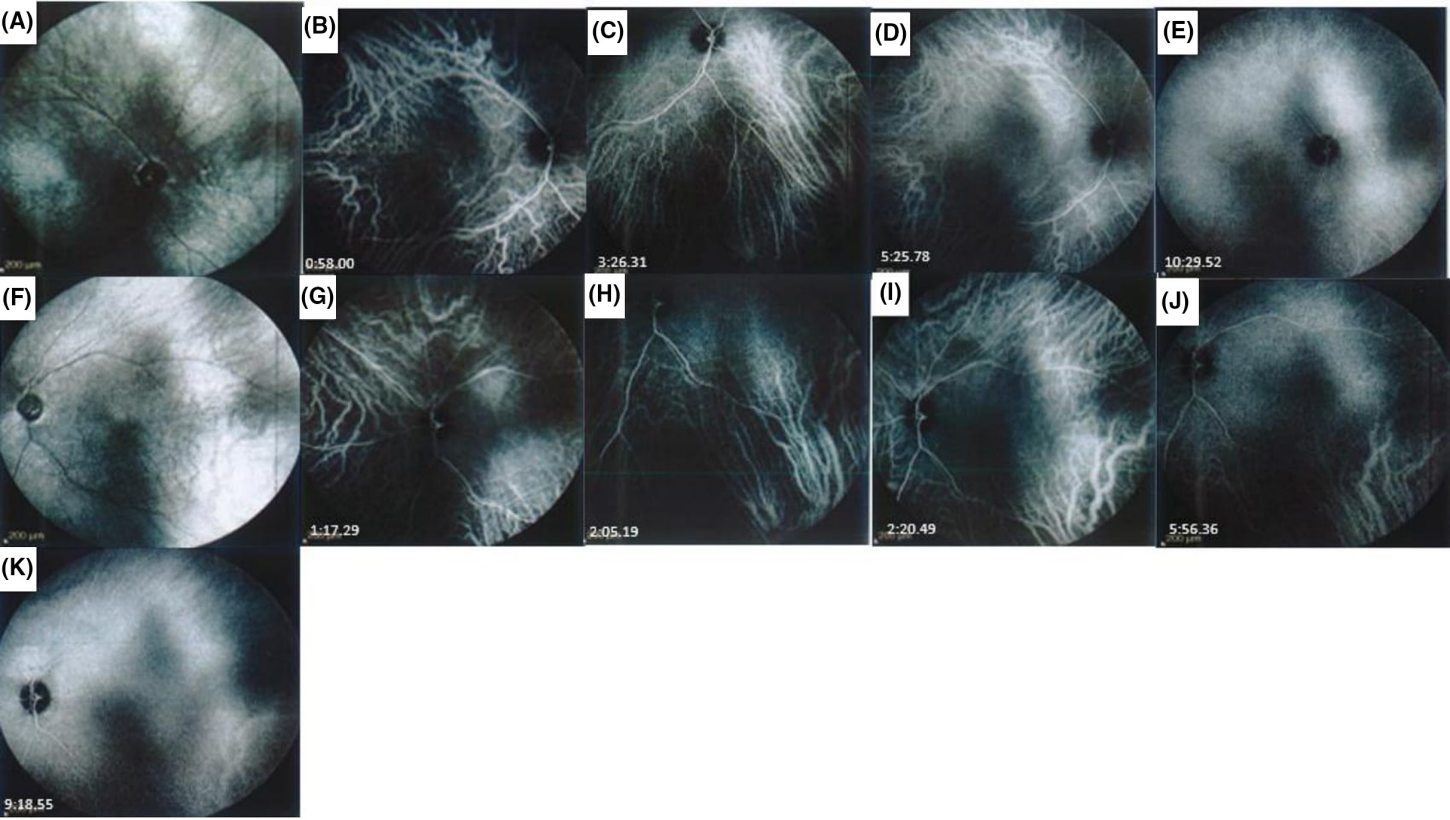
Indocyanine green angiography Of the RE (A–E) and the LE (F–K): multiple wedge‐shaped hypocyanescent areas indicative of choroidal ischemia.

**FIGURE 4:**
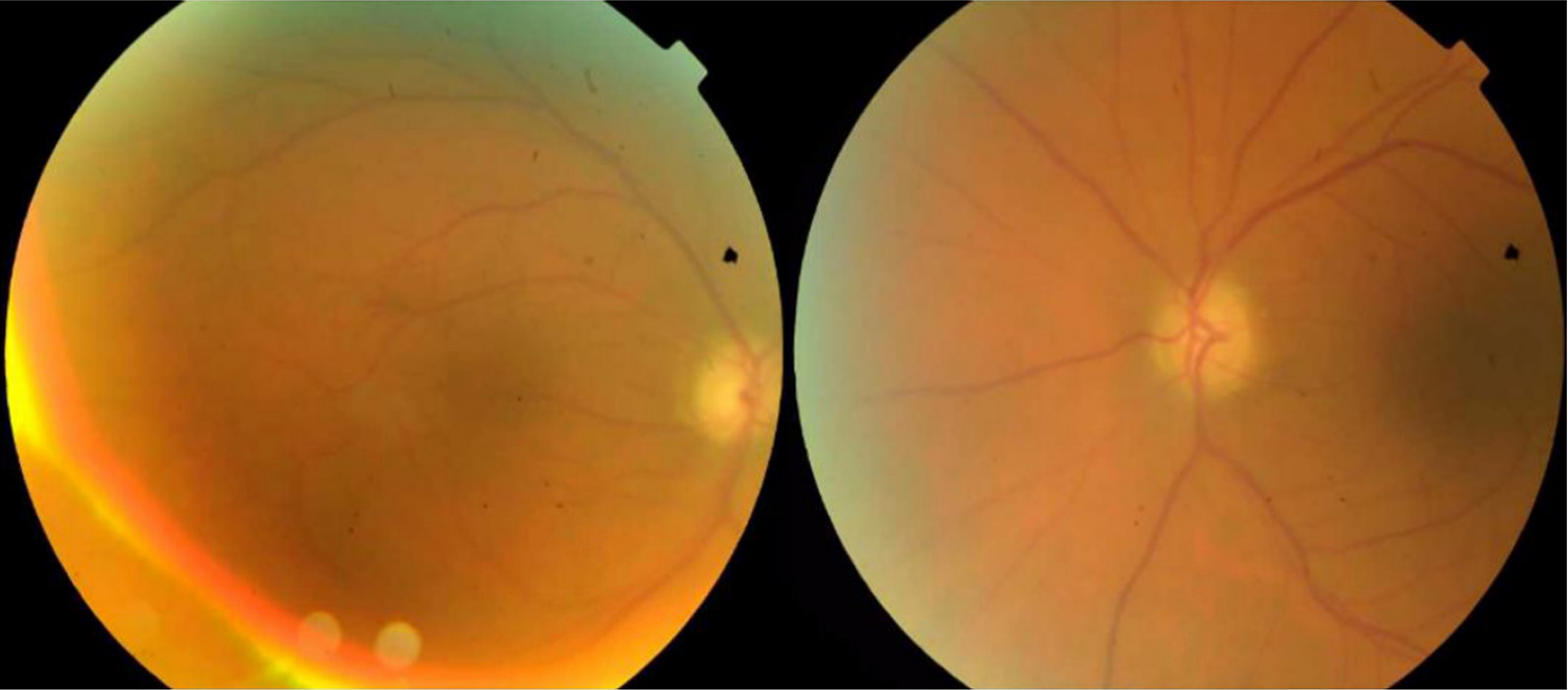
Color fundus photography: Resolved optic disk swelling in follow‐up examination

On examination, the best‐corrected visual acuity (BCVA) was light perception for the right eye (RE) and no light perception for the left eye (LE). Examination of the pupils revealed nonreactive pupils to light; therefore, the relative afferent pupillary defect was not assessable. Extraocular movements were normal. Intraocular pressure was measured 28 mmHg for the RE and 42 mmHg for the LE. Slit‐lamp examination findings indicated bilateral shallow anterior chambers (360° peripheral anterior synechiae (PAS) was found on gonioscopic examination), corneal microcystic edema, and nuclear sclerosis 1+ cataract in both eyes. A bilateral optic disk swelling was detected in fundus examination via undilated pupils. The patient was admitted, and after medical IOP reduction, peripheral laser iridotomy was done. Then, dilated fundoscopic examination performed, and bilateral optic disk swelling was found. Optic disks were small‐sized. Also, an intraretinal macular hemorrhage in the RE was seen (Figure 1A,D). Peripapillary retinal swelling was documented in retinal nerve fiber layer optical coherence tomography (RNFL OCT) (Figure 1B,E). Analysis of blood tests including complete blood test (CBC), erythrocyte sedimentation rate (ESR), and C‐reactive protein (CRP) was not remarkable. Prothrombin, partial thromboplastin time, antithrombin, protein C, proteinS, lupus anticoagulant, and lipid profile all resulted within the normal range. The HbA1c was 7.2%. According to optical biometry, axial length was 21.79 mm in the RE and 20.89 mm in the LE. The refraction of the RE was [+2.00  −1.00 @83] and [+6.75 −0.50 @ 13] for the LE. Increased choroidal thickness was noted in enhanced depth imaging OCT (EDI‐OCT), especially in the LE (Figure 1C,F). In fluorescein angiography, no sign of ischemia or peripheral vasculitis was detected (Figure 2). Indocyanine green angiography (ICG) confirmed severe choroidal ischemia (Figure 3). Color Doppler sonography of carotid and vertebral arteries and transthoracic echocardiography were unremarkable. No pathologic finding was reported in brain magnetic resonance imaging (MRI). Neurologist consult was done, and no evidence of giant cell arteritis and systemic vasculitis was found. Intravenous methylprednisolone (one gr daily) for three days was prescribed. In follow‐up examination, there was no improvement in visual acuity, IOP was controlled with topical medication, and optic disk swelling was resolved (Figure 4).

## 3 DISCUSSION

Ocular manifestations of SARS‐Cov‐2 in humans are not fully recognized. Previous reports indicated ophthalmic presentations from conjunctivitis and anterior uveitis to retinitis and optic neuritis.^3^

As shown in previous studies, there is an association between SARS‐Cov‐2 infection and hypercoagulability. The SARS‐Cov‐2 binds to the host cells by angiotensin‐converting enzyme (ACE)‐2 receptor(R). ACE‐2Rs are present in high density in the heart, lungs, arteries, and veins. So these organs are more vulnerable to the invasion of the pathogen. SARS‐Cov‐2 causes endothelial dysfunction by binding to ACE‐2Rs in retinal and choroidal microvasculature and leads to tissue ischemia, edema, and precoagulation state. Besides that, the systemic inflammatory response and cytokines release, activates the coagulation cascade which results in venous and arterial thromboembolic complications.^2^

COVID‐19 infection has the potential to cause damage to the choroid, retina, and optic nerve. Increased choroidal thickness, features of pachychoroid, and abnormal dilation of Haller's layer vessels are reported in patients with COVID‐19 infection in previous research.^4^ As discussed above, it seems that a disturbance in autoregulation of the RAAS system caused by the binding of SARS‐Cov‐2 to ACE‐2R results in endothelial dysfunction, increased vascular permeability, dilatation of retinal, and choroidal vessels. Changes in the retinal and optic nerve head microvasculature have been identified in previous research. A decrease in vessel density (VD) in the superficial and deep capillary plexus, as well as an increase in inner disk small vessel VD which can be associated with optic disk hyperemia and edema, has been documented.^5^, ^6^, ^7^ These alterations suggest that COVID‐19 infection puts patients at risk of choroidal and optic nerve disorders.

As mentioned, in our patient, bilateral painless vision loss occurred first, and pain was added after four days. According to the chronological order of symptoms, it seems that choroidal ischemia and optic nerve damage preceded the rise of IOP. On the contrary, while there are few studies evaluating whether acute elevated IOP affects retinal and choroidal circulation, some research supports the idea. In a study of patients with narrow anterior chambers, it was found that choroidal capillary blood flow decreased significantly when IOP elevated greater than 10 mm Hg, and retinal vessel density decreased significantly when IOP increased more than 20 mm Hg.^8^ Another study assessing the perfusion patterns of superficial and deep capillary plexus (SCP, DCP) and choriocapillaris (CC) following intravitreal injection of Bevacizumab found that DCP is affected the most, followed by CC and SCP.^9^ In our patient, we observed about 20 mm Hg rise in IOP, but despite severe choroidal ischemia in ICG, FA which shows retinal vasculature was not significantly affected. Moreover, EDI‐OCT showed reduced choroidal thickness in patients with choroidal ischemia caused by elevated IOP in the acute phase,^10^ while in the present case, the choroidal thickness has been increased. Due to the discussed reasons, we concluded that in our patient with predisposing factors for acute angle closure, including short axial length and hyperopia, increased choroidal thickness leads to anterior rotation of the ciliary body and contributes to pupillary block and angle closure attack.

Optic neuropathy may be caused by bilateral optic neuritis (ON) or by anterior ischemic optic neuropathy (AION). In the present case, there was no history of headache, scalp tenderness, fever, weight loss, and muscle weakness. Additionally, blood tests including CBC, ESR, and CRP were unremarkable. Also, systemic workup and neurologic evaluation found no evidence of systemic vasculitis or giant cell arteritis. Nonarteritic anterior ischemic optic neuropathy is caused by infarction of the short posterior ciliary arteries that supply the anterior portion of the optic nerve head. Although the exact pathophysiology of the condition is not recognized, some risk factors are noted in the literature including diabetes mellitus, hypercholesterolemia, smoking, anemia, and hypercoagulable state.^11^ In our patient, diabetes mellitus, which is a risk factor for AION, coexisting with COVID‐19 infection, which causes endothelial damage, hypoxia, and hypercoagulability, could put him at risk of NAION. Optic neuritis is often seen in young women, although atypical forms can be found in older populations. When the anterior part of the optic nerve is involved, painful eye movement is a common symptom in ON,^12^ which was absent in our patient. Another finding which is in favor of NAION in this case is the presence of sever choroidal ischemia. The cause of choroidal hypoperfusion may be secondary to SARS‐CoV‐2‐induced damages, including direct viral‐induced cell death, excessive inflammatory reactions accompanied by vascular inflammation and neutrophil infiltration.^13^

Nonarteritic anterior ischemic optic neuropathy is caused by hypoperfusion of the optic nerve. Our patient presented with bilateral NAION in the setting of COVID‐19 infection. There are three possible mechanisms for ocular complications in our patient: vascular endothelial damage, hypercoagulable state, and hypoxemia. At present, it is not possible to determine a causal or coincidental relationship between NAION and choroidal hypoperfusion, which occurred in described patient and COVID‐19 infection. But the purpose of this report was to shed light on the potential and multiplicity of ophthalmic presentation in a patient with COVID‐19 infection.

## AUTHOR CONTRIBUTIONS

**Seyed Hossein Ghavami Shahri:** Conceptualization; investigation; project administration; supervision. **Mojtaba Abrishami:** Supervision; writing – review and editing. **Helia Shayanfar:** Writing – review and editing. **Sahel Khazaei:** Conceptualization; investigation; resources; writing – original draft.

## FUNDING INFORMATION

This research received no specific grant from any funding agency in the public, commercial, or not‐for‐profit sectors.

## CONFLICT OF INTEREST

There are no conflicts of interest.

## CONSENT

Researchers ensure that they have obtained informed written consent from the patient for the publication of clinical examinations and retinal imaging.

## Data Availability

The data that support the findings of this study are available within the article. This manuscript has not been published or presented elsewhere in part or entirety and is not under consideration by another journal.
